# Past aridity and dust drove biodiversity crises and altered pollination in the ancient gymnosperm *Ephedra* (Gnetales)

**DOI:** 10.1111/brv.70019

**Published:** 2025-04-07

**Authors:** Natasha Barbolini, Niels Meijer, Carina Hoorn, Guillaume Dupont‐Nivet, Fang Han, Ashley Krüger, Qin Yuan, Alexander Rohrmann, Kristina Bolinder, Catarina Rydin

**Affiliations:** ^1^ Department of Ecology, Environment and Plant Sciences and Bolin Centre for Climate Research Stockholm University Stockholm SE‐106 91 Sweden; ^2^ Department of Biological Sciences University of Bergen and Bjerknes Centre for Climate Research Bergen 5020 Norway; ^3^ Senckenberg Biodiversity and Climate Research Centre (SBiK‐F) Senckenberganlage 25 Frankfurt am Main 60325 Germany; ^4^ Department of Ecosystem and Landscape Dynamics, Institute for Biodiversity and Ecosystem Dynamics University of Amsterdam Amsterdam 1098 XH The Netherlands; ^5^ Geosciences Rennes UMR‐CNRS, Université de Rennes Rennes 35042 France; ^6^ Helmholtz Centre Potsdam, GFZ German Research Centre for Geosciences Potsdam Germany; ^7^ School of Earth Sciences East China University of Technology Nanchang Jiangxi 330013 China; ^8^ Department of Palaeobiology Swedish Museum of Natural History Box 50007 Stockholm SE‐104 05 Sweden; ^9^ Key Laboratory of Comprehensive and Highly Efficient Utilization of Salt Lake Resources Qinghai Institute of Salt Lakes, Chinese Academy of Sciences Xining 810008 People's Republic of China; ^10^ Qinghai Provincial Key Laboratory of Geology and Environment of Salt Lakes Qinghai Institute of Salt Lakes, Chinese Academy of Sciences Xining 810008 People's Republic of China; ^11^ Institute of Geological Sciences Freie Universität Berlin Berlin 12249 Germany

**Keywords:** pollen, *Ephedripites*, ephedroid, entomophily, anemophily, climate change, loess, Eocene, Oligocene, fossil

## Abstract

The long‐term effects of present‐day climate change on pollination are unquantified. However, distinguishing climatic drivers of ancient changes in pollination could provide valuable insights into biotic responses to near‐future climate states. Herein, we show that pollination in a group of gymnosperm shrubs (*Ephedra* L., Gnetales) was irrevocably altered by the Cenozoic expansion of drylands on two different continents. In Asia, increased continentality during the mid‐Eocene drove aridification and strong, dust‐carrying storms that promoted a shift to prevailing wind pollination in the core clade of *Ephedra*. Surface uplift in the North American interior together with global cooling caused the expansion of aeolian deposition and placed similar evolutionary pressures on ephedras there, beginning in the latest Eocene and continuing across the Eocene–Oligocene transition (EOT). These climatic changes fundamentally altered the abundance and evolution of this ancient plant lineage on both continents and determined pollination mechanisms in the core clade of *Ephedra* today. Based on fossil evidence, this review demonstrates how climate change may have major and permanent impacts on plant–pollinator networks, as well as demonstrates possible evolutionary consequences of near‐future climate scenarios for which we have no modern analogue.

## I. INTRODUCTION

Unprecedented global climate change has occurred during the Anthropocene, affecting ecosystems, human infrastructure, agriculture, and pollination (IPCC, 2023). Pollination is an essential service without which all major terrestrial ecosystems would not survive, but our knowledge and response actions have not kept up with the threats it faces (Potts & Imperatriz‐Fonseca *et al*., 2016). Climate‐driven shifts in phenology and ranges are causing mismatches between many plants and their pollinators, with too few studies available to draw broad conclusions on the consequences for plant–pollinator networks (Settele, Bishop & Potts, 2016).

Reconstructing pollination systems of the past can provide valuable information for understanding how plant–pollinator networks could respond to climate change, particularly since Earth's climate state is rapidly moving to atmospheric carbon dioxide concentrations not seen for ~15 million years (Ma), and perhaps as much as ~50 Ma (Burke *et al*., 2018; Steinthorsdottir *et al*., 2021). An additional advantage of studies on the pre‐human past is that it is difficult to disentangle contemporary climate change effects on pollination from other interacting, human‐caused drivers such as land degradation and pesticides (Potts & Ngo et al., 2016; Knight *et al*., 2018). While no palaeoclimate can be a perfect equivalent for future climate scenarios, deep‐time fossil archives are playing a crucial role in understanding the sensitivity of Earth systems to global change (Burke *et al*., 2018; Steinthorsdottir *et al*., 2021).

Fossils of *Ephedra* (Gnetales) are preserved in sedimentary archives around the world, representing a group of ancient gymnosperm shrubs with a very long history (at least ~125 Ma) that still exist today (summarised in Friis, Crane & Pedersen, 2011; also see Ickert‐Bond & Renner, 2016; Rydin & Hoorn, 2016). They are remarkably resilient, having survived at least one global mass extinction, as well as numerous other hyperthermal and cooling events. Ecological experimentation and observations, and aerodynamic studies indicate that at least two distinctly different pollination mechanisms occur today in *Ephedra*, and evolutionary shift(s) in pollination are hypothesised to have taken place in the past (Rydin & Bolinder, 2015; Bolinder *et al*., 2016a). This makes these plants with a near‐global distribution an ideal group for studying how and why plant–pollinator networks could change in response to future climate scenarios, for which we have no modern analogue.

**Fig. 1:**
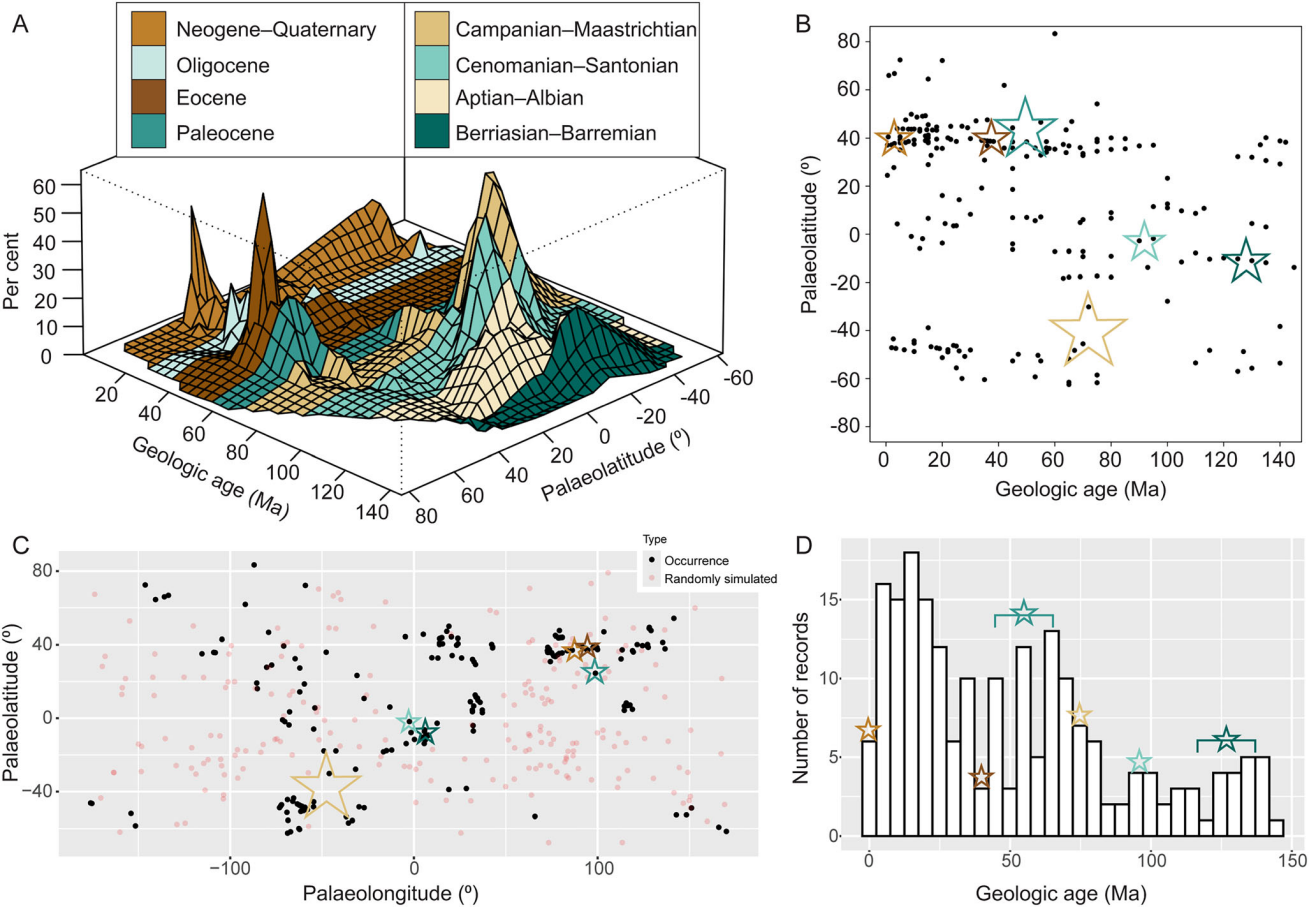
(A) Global distribution of ephedroids through time based on occurrence of dispersed ephedroid pollen in sediments from the Early Cretaceous to the present. Plotted points each represent a reported percentage of ephedroid pollen, or absence thereof, from a fossil locality described in a palynoflora or palynological assemblage. (B) The 207 underlying data points (Table S1) are relatively evenly spread over the investigated time–space. (C) Scatter plot of record co‐ordinates (black circles) for visual assessment of clustering compared with randomly simulated points at the same density (red points). (D) Histogram plot of number of records through time. In C and D, the peaks of ephedroid abundance identified in A are represented by coloured stars, illustrating that these peaks are not influenced by underlying spatio‐temporal biases in the data set. The stars are coloured in the respective colours of the chronostratigraphic periods shown in A and the sizes of stars span the records that generate that particular peak, with no implication on peak size.

The oldest unambiguous ephedroid fossils belonging to the Gnetales, as the order is defined today, are mostly from low palaeolatitudes where they appear to have been abundant in the Early Cretaceous. This is documented by an increasing number of macrofossil discoveries as well as microfossil evidence (for references see online Supporting Information, Table S1). Pre‐Cretaceous ephedroids have been reported in the literature (see for example Wang, 2004) but the exact relationship of these intriguing fossils to the present‐day Gnetales remains controversial [see Coiro *et al*. (2022) for further discussion]. Accordingly, here we focus on the Cretaceous and Cenozoic record of ephedroids, mostly comprising microfossil data (Fig. 1), which shows that ancient climate change and/or competition with early angiosperms may have pushed ephedroids to refugia at higher palaeolatitudes from the mid‐Cretaceous onwards. Ephedroid pollen has been widely used as a climate proxy to indicate a dry palaeoenvironment (e.g. Sun & Wang, 2005), but precise reasons for the repeated and globally occurring peaks and crashes in ephedroid diversity and/or abundance (Fig. 1) have never been rigorously assessed, hindering palaeoclimate reconstructions and our understanding of past pollination syndromes in ephedroids.

Surprisingly, ephedroids seem to have been relatively unaffected by the enormous ecological upheavals that took place during the Cretaceous–Paleogene (K–Pg) mass extinction 66 million years ago (Fig. 1). Our initial overview instead indicates major fluctuations in the presence of fossil ephedroid pollen before the K–Pg boundary (in low palaeolatitudes and the Southern Hemisphere), and long after the K–Pg boundary in the Northern Hemisphere (Fig. 1). Both Central Asia and North America show major changes in ephedroid pollen types during the late Paleogene (Tables S2 and S3). Based on this evidence, here we review and explore records of ephedroids in these regions from the Cretaceous to the present, providing the opportunity to test the hypothesis that drastic climate changes, from greenhouse to icehouse states, may have driven biodiversity crises and shifts in pollination syndrome. We assess the role that climate and pollination played in the survival of the family to the present and the significance of our findings for predictions of short‐ and long‐term consequences for plant–pollinator networks under a changing climate.

## II. FEATURES OF INSECT‐ *VERSUS* WIND‐POLLINATED EPHEDROID POLLEN

**Fig. 2:**
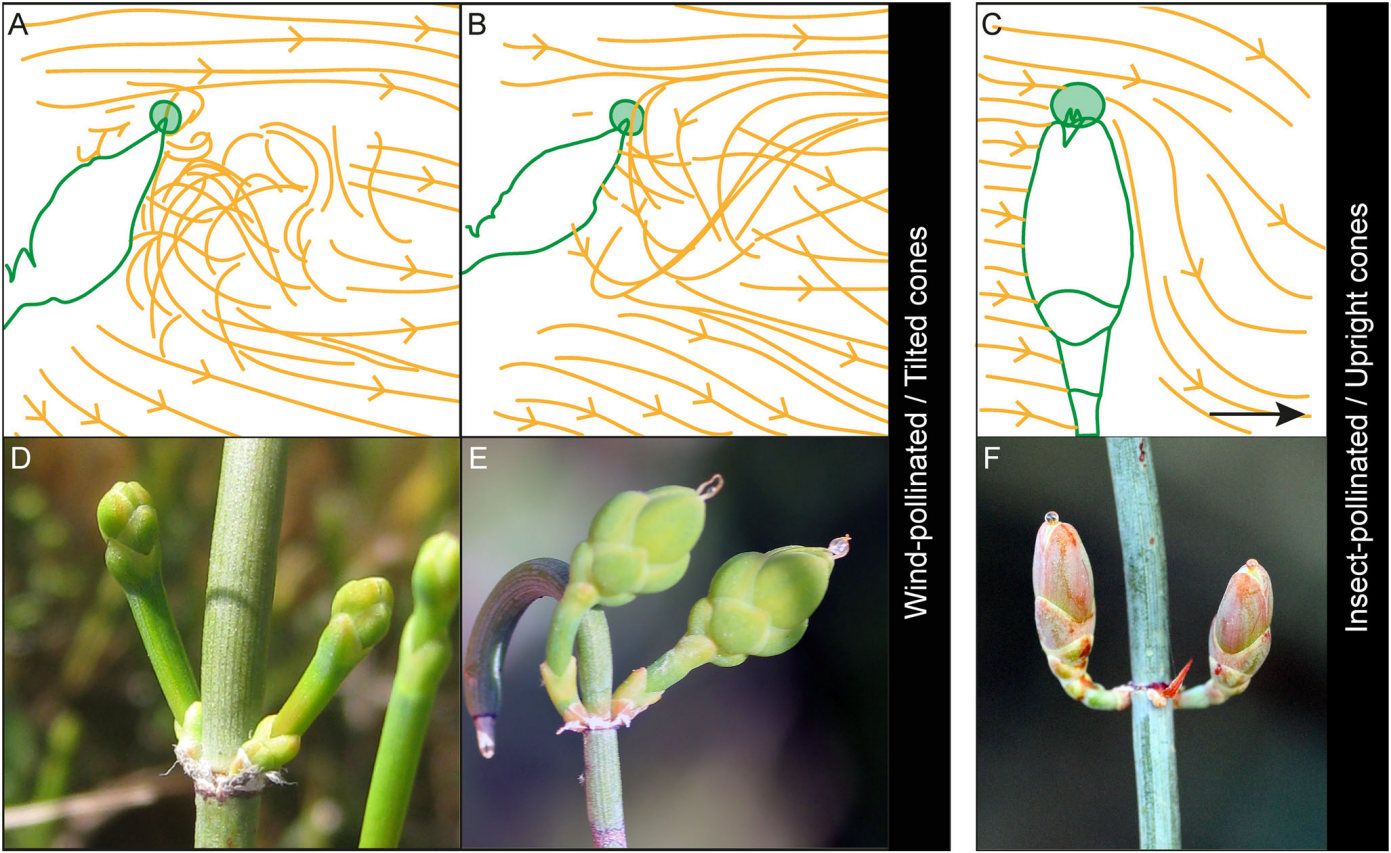
Aerodynamics and pollen reception in female ephedroid cones. (A–C) Computer simulations of pollen grain (shown in orange) aerodynamics around ovules with pollination drops (shown in green) of three extant species *Ephedra trifurca* Torr. ex S.Wats. (A), *E. nevadensis* S.Wats. (B), and *E. foeminea* Forssk. (C) in lateral view (modified after Niklas & Buchmann, 1987; Bolinder *et al*., 2015; Niklas, 2015). Airflow direction is from left to right in all panels. (D–F) Female cones of *Ephedra*. Wind‐pollinated species of *Ephedra*, such as *E. distachya* L. (D, E) often have a tilted orientation on the plant and they create an airflow pattern that enhances their capacity to capture pollen of the correct size. The female cones of the insect‐pollinated *E. foeminea* (F) lack these features, and its female cones are upright, born on bent pedicels (F). Photographs: Kristina Bolinder.

Almost all extant *Ephedra* species are wind‐pollinated, and have morphological adaptations of pollen to enhance the process aerodynamically, as well as adaptations related to pollen reception. Wind‐pollinated ephedras create an air flow pattern around the female cone that enhances capture of conspecific pollen, directing it towards the pollination drops (Fig. 2A, B; Niklas, Buchmann & Kerchner, 1986; Niklas & Kerchner, 1986; Niklas & Buchmann, 1987; Buchmann, O'Rourke & Niklas, 1989; Bolinder, Niklas & Rydin, 2015; Niklas, 2015). These specialised properties for pollen reception are absent in the sole living insect‐pollinated species of *Ephedra*, *E. foeminea* Forssk. (Fig. 2C; Bolinder *et al*., 2015). One reason for this difference among species in aerodynamic properties is the orientation of the pollen‐receptive surfaces in relation to the direction of the airflow (Niklas, 1985; Bolinder *et al*., 2015); female cones of wind‐pollinated species of *Ephedra* are typically tilted with respect to gravity (Fig. 2D, E), whereas those of the insect‐pollinated *E. foeminea* are upright (Fig. 2F). In addition, there are other differences between insect‐pollinated and wind‐pollinated ephedran species: the insect‐pollinated *E. foeminea* has morphologically bisexual male cones that attract pollinators using pollination drops; and the derived type of ephedroid pollen (see below) is known only in wind‐pollinated species. Morphological differentiation in modern and extinct *Ephedra* is generally low (e.g. Rydin, Pedersen & Friis, 2004; Rydin, Khodabandeh & Endress, 2010), yet these examples demonstrate that small morphological differences can have substantial functional consequences. Unfortunately, variation in female cone morphology is challenging to observe in the fossil record: while a diverse record of ephedroid plant macrofossil remains (as well as pollen) have been reported from the Early Cretaceous, the fossil record of *Ephedra* for the Late Cretaceous and Cenozoic is based solely on pollen (Rydin & Hoorn, 2016). Accordingly, herein we focus on ephedroid pollen.

**Fig. 3:**
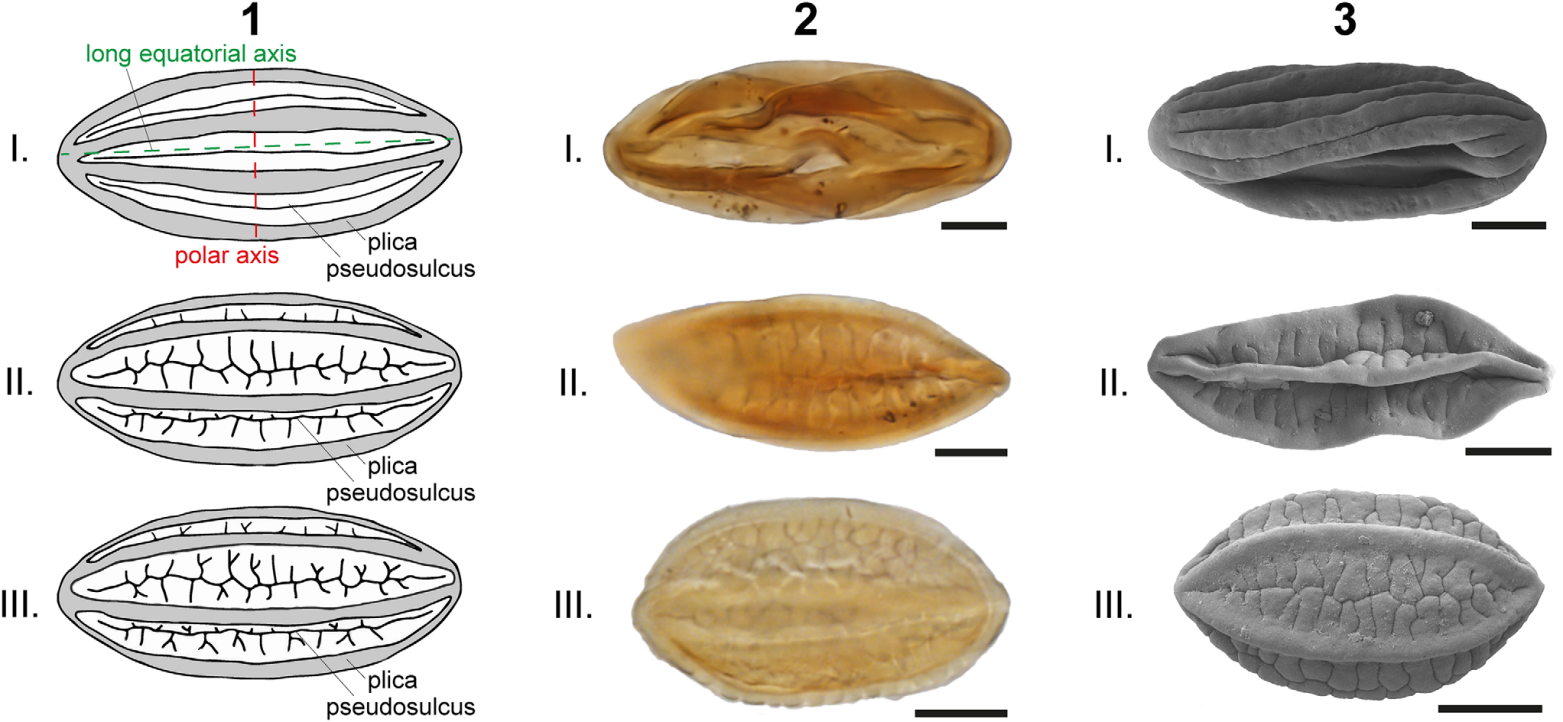
Morphology and pseudosulcus branching in *Ephedra* pollen. (1) Illustrations of the ancestral (type I) and derived (type II: first‐order branching; type III: second‐order branching) patterns, which are present in both living and fossil grains (drawn after an original in Bolinder *et al*., 2016b). (2) Light microscope (LM) and (3) scanning electron microscope (SEM) images of *Ephedripites* (fossil *Ephedra* pollen) from the Eocene of Central Asia; 2 & 3 (I.), *E. lanceolatus* Zhu & Wu (1985), 2 & 3 (II.), *E. fusiformis* Habib (1970), 2 & 3 (III.), *E. fushunensis* Sung & Tsao (1978). LM and SEM photos of each morphospecies depict different pollen grains. LM photos: Natasha Barbolini; SEM photos: Fang Han.

Ephedroid pollen (extant and fossil) can be classified into two types: ancestral and derived morphotypes (Bolinder *et al*., 2016b; Han *et al*., 2016). The ancestral pollen type, known at least since the Early Cretaceous and identified *in situ* in ephedroid seeds from this epoch (Rydin *et al*., 2004), has numerous plicae interspersed with unbranched pseudosulci (Fig. 3: type I). The derived pollen type, not known in the fossil record until the Late Cretaceous (Rydin *et al*., 2021), has on average fewer plicae than the ancestral type and occurs in two forms, either with first‐order side branches on the pseudosulci (Fig. 3: type II) or with first‐ and second‐order side branches (Fig. 3: type III). On this basis, the palynological form genus *Ephedripites* is divided into the two subgenera *Ephedripites* subgen. *Ephedripites* and *Ephedripites* subgen. *Distachyapites* (following Han *et al*., 2016), which correspond to the ancestral and derived types respectively (Bolinder *et al*., 2016b).

Pollen morphology, particularly pseudosulcus branching, is linked to pollination syndrome in living *Ephedra*. The derived pollen type is, as far as is known, produced exclusively by wind‐pollinated plants (Bolinder *et al*., 2015; Niklas, 2015). Bolinder *et al*. (2015) showed that pseudosulcus branching facilitates wind pollination in several ways, which together contribute to an ability to remain airborne for longer. The ultrastructure of the pollen wall also affects its aerodynamic properties and may be linked to pollination syndrome. The insect‐pollinated *E. foeminea* produces the ancestral type of pollen with a small infratectum compared to pollen of the derived type (Bolinder *et al*., 2015). This is likely another reason why these grains stay airborne for shorter periods compared to those of the sympatric wind‐pollinated species *E. distachya* L. (Bolinder *et al*., 2016a). The ancestral pollen type in *E. foeminea* was also shown to have a tendency to form clumps, which further inhibits their ability to remain airborne (Bolinder *et al*., 2015, 2016a).

Ancestral *Ephedripites* pollen grains from the Early to mid‐Cretaceous possess a similar ultrastructure to that of *E. foeminea* pollen, with a thick tectum and small infratectum (Trevisan, 1980; Bolinder *et al*., 2015), suggesting they may have been insect‐pollinated. This assumption is supported by observations of ovular support and strong thickening of the micropylar tube both in fossils and living species (e.g. Rydin & Friis, 2010; Rydin *et al*., 2010). These traits could protect the micropylar tube from bending or breakage when supporting the weight of visiting insects. Strong thickening of the micropylar tube is retained in several modern species of *Ephedra* that produce pollen of the ancestral type (e.g. *E. foeminea* and *E. aphylla* Forssk.) but has been lost in modern wind‐pollinated species of *Ephedra* with the derived type of pollen, such as *E. distachya* (Rydin *et al*., 2010).

At least one presumed wind‐pollinated species [the North American *E. trifurca* Torrey ex S. Watson (Niklas *et al*., 1986; Niklas & Kerchner, 1986; Buchmann *et al*., 1989)] produces the ancestral pollen type, thus obscuring any correlation between pollen type and pollination syndrome, at least for the ancestral pollen type. Interestingly however, Niklas & Buchmann (1987) describe *E. trifurca* as “more of an aerodynamic generalist” compared to the sympatric wind‐pollinated *E. nevadensis* S.Watson with its derived type of pollen. Similar claims of a generalist pollination system utilising both abiotic and biotic vectors for pollen transfer have been made for two Mediterranean species with the ancestral type of pollen, *E. aphylla* (Meeuse *et al*., 1990) and *E. fragilis* Desf. (Celedón‐Neghme, Santamaría & González‐Teuber, 2016), and investigations of additional species are warranted. While the presence of current or former wind‐pollinated species that produce the ancestral type of pollen cannot be excluded, current knowledge suggests that the ancestral ephedran pollen type is and was mostly linked to insect pollination, although sometimes in combination with abiotic pollen transfer in an ambophilous system.

One argument sometimes used to suggest that Cretaceous ephedras were wind‐pollinated, rather than insect‐pollinated, is the comparatively strong presence of ephedroid pollen in many fossil archives. The extant wind‐pollinated species *E. distachya* generally produces much more pollen than the insect‐pollinated *E. foeminea* (Bolinder *et al*., 2015, 2016b; Hofmann, Roberts & Seyfullah, 2022). However, the extinct entomophilous conifer family Cheirolepidiaceae (Pocock, Vasanthy & Venkatachala, 1990; Krassilov, Zherikhin & Rasnitsyn, 1997; Krassilov, Rasnitsyn & Afonin, 2007; Labandeira, Kvaček & Mostovski, 2007; Ren *et al*., 2009) exemplifies that such a pattern is not universal. The Cheirolepidiaceae produced distinctive pollen (*Classopollis* Pflug) that is at least as abundant or more so than that of known abiotically pollinated plants in many deposits globally (e.g. Archangelsky & Gamerro, 1967; Vakhrameev, 1981; Wang, Mosbrugger & Zhang, 2005; Barreda *et al*., 2012; Tosolini *et al*., 2015; Zhang *et al*., 2019). Both cheirolepidiaceous and gnetalean pollen have been found in the gut contents of several Mesozoic insect lineages (Krassilov *et al*., 1997, 2007; Labandeira *et al*., 2007), and must have served as a nutritional source and/or pollination reward.

Thus, based on these data and observations of fossil and living species, it has been suggested that insect pollination was more common in *Ephedra* in the past than it is today, perhaps even originally the dominant mode of pollination in the genus (Bolinder *et al*., 2015, 2016b). A variety of new (derived) ephedroid pollen types (see Fig. 3) not known before the Turonian–Coniacian boundary of the Late Cretaceous (Steeves & Barghoorn, 1959; Rydin *et al*., 2021) became common in the Paleogene (from *ca*. 58 Ma) and largely replace the ancestral pollen type in many Cenozoic strata (Han *et al*., 2016). The novel pollen type shares the characters of wind‐pollinated *Ephedra* species today, and it was thus hypothesised that an evolutionary shift to wind pollination occurred relatively rapidly in *Ephedra* (Bolinder *et al*., 2015, 2016a; Hofmann *et al*., 2022). In these studies, the catastrophic asteroid impact at the K–Pg boundary was tentatively suggested to have caused this evolutionary transition (e.g. Bolinder *et al*., 2016a) but a rigorous temporal and causal framework for this hypothesis was not established. Therefore, herein we show that Cenozoic climate changes appear to have played a primary role in driving pollination shifts in the ephedran lineage.

## III. METHODS

To obtain an initial overview of ephedran abundance changes through time, a database of the global distribution of *Ephedripites* (i.e. pollen of the genus *Ephedra*) from the Early Cretaceous to the present was assembled, following the approach of Crane & Lidgard (1989) (Table S1). In this data set, pollen was not separated into morphospecies (i.e. species based solely on morphology), since many previously published studies did not provide this information. While this data set cannot therefore provide information about pollination changes in ephedras through time, it can be used to identify overarching patterns and particular geographical areas and time periods of interest, which we could then focus on using a smaller but more detailed data set (see Sections IV and V). The data were made continuous by a linear interpolation using the Akima package, and a 3D plot (Fig. 1A) was constructed using the *persp* function, both in R v. 4.3.1 (R Development Core Team, 2023).

To test for spatial and temporal biases, we used the *dynamicSDM* package for R (Dobson *et al*., 2023) to perform a chi‐squared test (Greenwood & Nikulin, 1996) to assess temporal sampling bias, and a *t*‐test following the nearest neighbour index (Clark & Evans, 1954) to assess spatial bias (see footnote to Table S1 for results). While the global fossil ephedroid record is biased in space and time, a scatter plot of record co‐ordinates for visual assessment of clustering (Fig. 1C) and a histogram plot of record frequency through time (Fig. 1D) show that the record is biased towards the late Neogene and to continental landmasses (accounting for changing palaeogeography through time), as expected for fossil records of a land plant. Considering that all palaeontological data sets contain varying degrees of taphonomic and collecting bias, we sought to identify whether the abundance peaks in Fig. 1A are influenced by the inherent bias of the fossil record. Plotting these abundance peaks (represented by stars in Fig. 1B–D) onto the spatiotemporal spread of data points, it is clear that these peaks are not biased by their location in time (Fig. 1B), space (Fig. 1C) or the number of records collected (Fig. 1D).

This overview of repeated rises and crashes in global ephedroid abundance through time (Fig. 1B) was then used to focus attention on particular areas of interest (in time and space), where we can use more data, often with better and/or independent age control and better possibilities to identify the grains to morphospecies. Using these criteria, we assembled an Early Cretaceous–Holocene compilation of ephedroid pollen (Tables S2 and S3) restricted to the genus *Ephedripites* Bolkhovitina (1953) ex Potonié (1958) emend. Krutzsch (1961) and separated into morphospecies where possible (following Han *et al*., 2016) or pollen types (Fig. 3). The genus *Ephedripites* has sometimes been called *Ephedra* or *Equisetosporites* by various authors. When the latter name was used in a publication, identification was confirmed visually as many morphospecies of *Equisetosporites* are not ephedroid pollen. Similarly, some studies did not distinguish to species or type of *Ephedripites*, and in such cases identification was conducted based on photographs and/or descriptive text wherever possible.

**Fig. 4:**
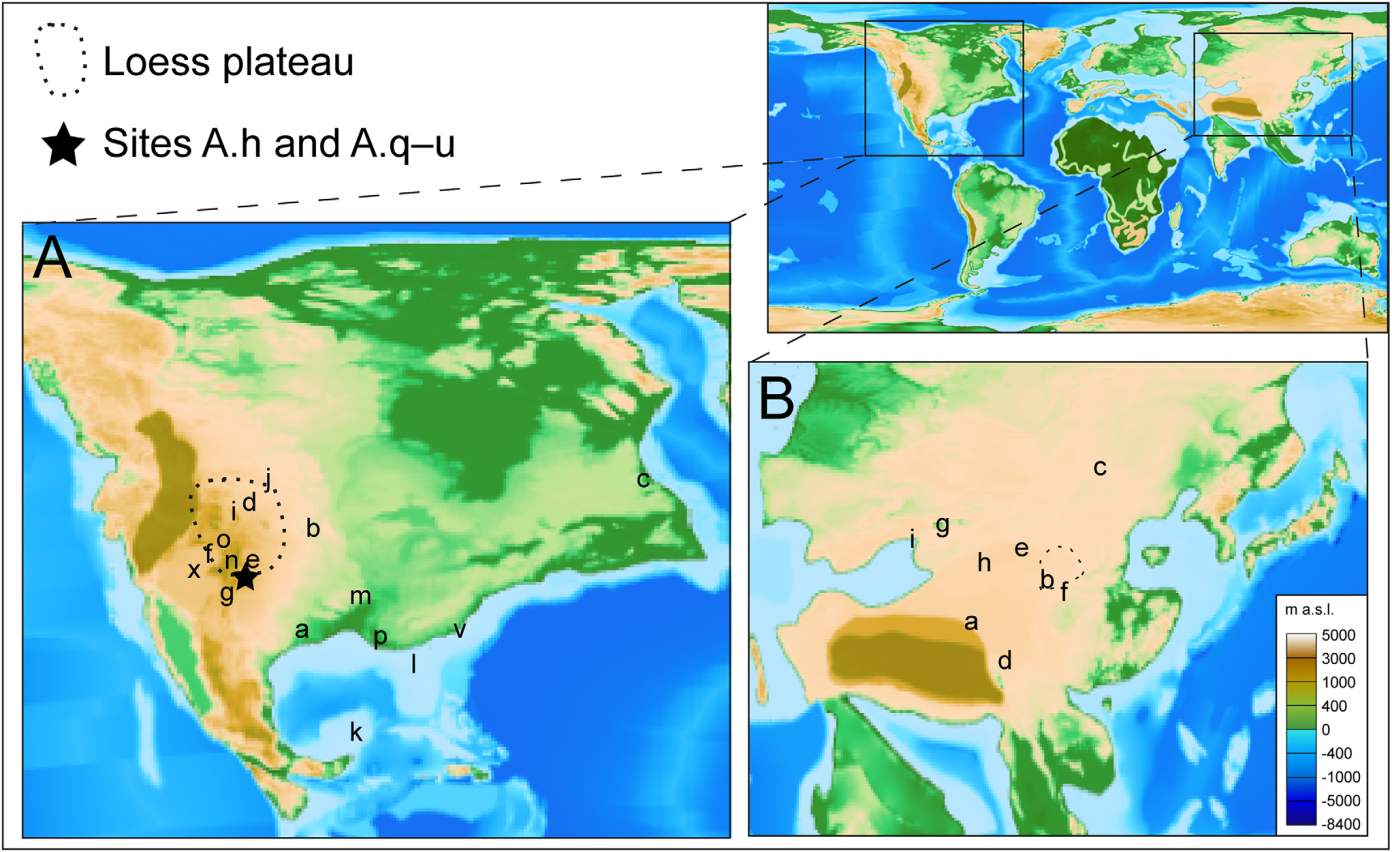
Location of the study areas in North America and Central Asia. (A, B) Study sites (letters) detailed in Tables S2 and S3. Loess plateau reconstructions are derived from Fan *et al*. (2020) and Meijer *et al*. (2021). The palaeogeographic reconstruction (at 40 Ma) is from Poblete *et al*. (2021); the data and map were downloaded from the Paleoenvironment map website (https://map.paleoenvironment.eu) on 08 June 2022, using the following parameters: reconstruction time: 40 Ma, Indo‐Asia tectonic model: Double Collision Greater India Basin, palaeogeography: Turgai Strait open. Colours indicate meters above sea level (m a.s.l.). Note that study sites range in age from Cretaceous to Oligocene.

Published data from North America (Table S2) and Central Asia (Table S3) were compiled together with an updated and expanded record from the well‐dated and time‐extensive Xining Basin in Central Asia (N. Barbolini, H. van den Hil, C. Hoorn, A. Woutersen, N. Meijer, G. Dupont‐Nivet, F. Han, A. Rohrmann & C. Rydin, in preparation). We used this data set to study fluctuations in diversity and relative abundance of *Ephedra* through time on both continents, and to infer pollination changes (Fig. 4). Two main limitations exist for this data set, namely, that (*i*) the number of pollen morphospecies in any stratigraphic horizon almost certainly does not equal a direct count of actual plant species living at that time, and (*ii*) differing amounts of pollen produced by wind‐ *versus* insect‐pollinated plants may introduce a bias into the interpretation of relative pollen counts. To mitigate the first limitation, we assume that a relative change in pollen abundance broadly corresponds to an overall change in regional plant diversity over a longer period of time (i.e. multiple seasons). To reduce possible effects of the second limitation, we use both proportions and raw counts when comparing the fossil pollen record of ancestral (likely insect‐pollinated or mixed pollination strategies) *versus* derived (likely wind‐pollinated) ephedroid pollen.

For the Asian data, included samples were counted by only two authors (F. Han and N. Barbolini) and followed the same method, controlling for raw counts and using the morphospecies figured in Han *et al*. (2016). We thus considered it appropriate to link relative abundances with climatic events. The North American data were derived from published studies by multiple authors, generating more uncertainty in how these data can be compared. Accordingly, for the North American data set we primarily use the Cretaceous absence and Cenozoic appearance of the derived type of pollen and of second‐order pseudosulcus branching (which are confirmed by the same pattern globally) rather than the relative abundances of raw counts to infer the onset of dust storms and aridity in the middle‐late Eocene of North America. Furthermore, based on all records, it is clear that the first presence of the derived type globally is within the Cretaceous Raritan Formation in North America, whereas the ancestral type is clearly older.

## IV. FOSSIL DOCUMENTATION OF EPHEDROID POLLEN IN THE NORTHERN HEMISPHERE THROUGH TIME

### (1) North American ephedroid pollen

Pollen records from the USA and Canada show that the ancestral type of *Ephedripites* (unbranched pseudosulci; Fig. 3: type I) was already common by the late Early Cretaceous. It is present in multiple localities across western North America and often identified as “*Equisetosporites ovatus”* in the literature (Table S2). The ancestral pollen type is commonly present together with *Gnetaceaepollenites* F. Thiergart (1938), which is restricted to the Late Cretaceous─Paleocene; we agree with Frederiksen (1980) that the few grains recovered from the late Eocene of Alabama are almost certainly reworked (Table S2). The derived (branched pseudosulci) pollen type first appears in the early Late Cretaceous (Turonian; Steeves & Barghoorn, 1959; locality c of Fig. 4A), but remains rare in North America until the middle Eocene (dating not precise enough to refine this to either the Lutetian or the Bartonian; Table S2). The derived type with first‐ and second‐order psuedosulci branching (Fig. 3: type III) appears infrequently from the middle Eocene (Lutetian) but only becomes common in the late Eocene (Bartonian) in Alabama and South Carolina, and the latest Eocene in Colorado (Table S2).

As the derived types of *Ephedripites* become more common, so the ancestral type becomes rarer, and during the middle–late Eocene the latter is not found in samples where the derived types are present (localities l–o of Fig. 4A). In the late Eocene of Alabama and the late Oligocene of South Carolina, a single ancestral species [*E. (E.) hungaricus* Nagy (1963)] is infrequently recorded (Table S2). Latest Eocene and Oligocene floras (34.1–26.9 Ma) from central and southwestern Colorado (Leopold & Zaborac‐Reed, 2019) contain a single ancestral type, *E. (E.) torreyana* Steeves & Barghoorn (1959), in very low proportions of the total pollen counts (Table S2). In the late Oligocene, the derived type comprises 9% of an assemblage from western Utah, compared with 1.5% for the ancestral type (Table S2). Thus, the derived type of pollen is clearly much more abundant than the ancestral type from the middle Eocene onwards.

### (2) Central Asian ephedroid pollen

The Central Asian region comprises modern‐day Mongolia, northern and western China, and northwestern (geographic) Tibet (Barbolini *et al*., 2020). Ephedroid pollen is reported infrequently and in low amounts from this region during the Early Cretaceous (Table S3). By the Late Cretaceous, the derived pollen types with first‐ and second‐order branching became common and Central Asia hosted a high ephedroid diversity (>100 morphospecies) during the Late Cretaceous–Cenozoic (b in Table S3; Wang *et al*., 1990b; Song *et al*., 1999). Many new morphospecies emerged in the Eocene, particularly the derived types (Han *et al*., 2016), and *Ephedra* evidently formed a significant component of Central Asian vegetation for tens of millions of years (Wang *et al*., 1990b; Zhang & Zhan, 1991; Sun & Wang, 2005; Hoorn *et al*., 2012; Ivarsson, 2013; Bosboom *et al*., 2014; Barbolini *et al*., 2020). Pollen grains of all three types (I–III; Fig. 3) are present in most samples from the high‐resolution Xining Basin record in northeastern Tibet (locality b in Fig. 4B), but their abundance and diversity change markedly through the late Paleocene to early Oligocene (Table S3).

**Fig. 5:**
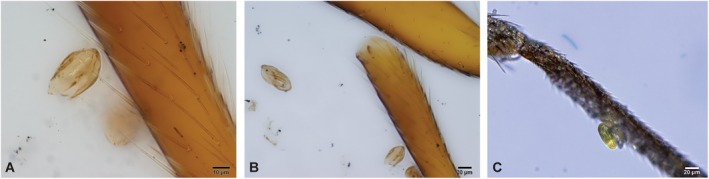
Ancestral‐type *Ephedra* pollen on arthropod legs. (A) Eocene (~39.9 Ma) *Ephedripites* pollen grain of the ancestral type, attached to a seta of an arthropod (sample TF P4‐18). (B) Same image at lower magnification, with a grain of another ancestral ephedroid pollen type, *Steevesipollenites major* Zhang & Zhang (1991), visible to the left. (C) Modern pollen grain of ancestral‐type *Ephedra foeminea* on the leg of an insect of Sciaridae (Bolinder *et al*., 2016a). Photographs A and B: Natasha Barbolini.

In general, the ancestral type is more common in the early Eocene, spikes in the middle Eocene, and decreases in the late Eocene to become rare thereafter (localities b, d and e of Fig. 4B; Table S3). One of our Eocene samples from Xining (dated to ~39.9 Ma with an upper error limit of 40.2 Ma) contained numerous disassociated arthropod (insect/arachnid) parts, one with setae bearing trapped ephedroid pollen grains of the ancestral type (Fig. 5A, B). Note, however, that all samples underwent typical palynological processing with disaggregation, acid digestion, and centrifugation, meaning that this association may simply be taphonomic. However, this sample contained both the highest abundance and diversity of ancestral types of ephedroid pollen [i.e. pollen type I (Fig. 3) that is produced by all species known to be insect‐pollinated or ambophilous (Rydin & Bolinder, 2015; Bolinder *et al*., 2016, 2016; Meeuse *et al*., 1990; Celedón‐Neghme *et al*., 2016), but possibly also some wind‐pollinated species] out of all samples in the detailed Xining record (N. Barbolini, H. van den Hil, C. Hoorn, A. Woutersen, N. Meijer, G. Dupont‐Nivet, F. Han, A. Rohrmann & C. Rydin, in preparation). In addition, the pollen is attached to the setae in the same way as modern pollen produced by the insect‐pollinated *Ephedra foeminea* attaches to the setae of its vector (Fig. 5C; Bolinder *et al*., 2016a). The occurrence of the ancestral ephedroid pollen type (Fig. 3: type I) subsequently rapidly declines, concurrently with the middle–late Eocene rise in abundance of derived ephedroid pollen.

First‐ and second order‐branched (derived) pollen (Fig. 3: types II and III) become increasingly abundant and more diverse through the Eocene up until the Eocene–Oligocene Transition (Table S3). Early Oligocene samples may contain rare pollen grains of the ancestral type along with those of the derived type but late Oligocene samples lack the ancestral type and pollen with second‐order branching appears rare in the entire epoch (localities b and e–g of Fig. 4B; Table S3). The same is true for Miocene, Pliocene and Quaternary samples; pollen grains of the ancestral type are absent and of most of the derived type of pollen lacks second‐order branching, although the latter is present in Miocene sediments from the Qaidam Basin (locality h of Fig. 4B; Table S3). It should be noted, however, that some studies in the literature did not distinguish between the different ephedroid morphospecies (see also Section III and Tables S2 and S3). Where possible, we reassessed the type more specifically, based on photographs and/or descriptions in the original work.

## V. EPHEDROID POLLEN DIVERSITY, POLLINATION AND CLIMATE CHANGE

**Fig. 6:**
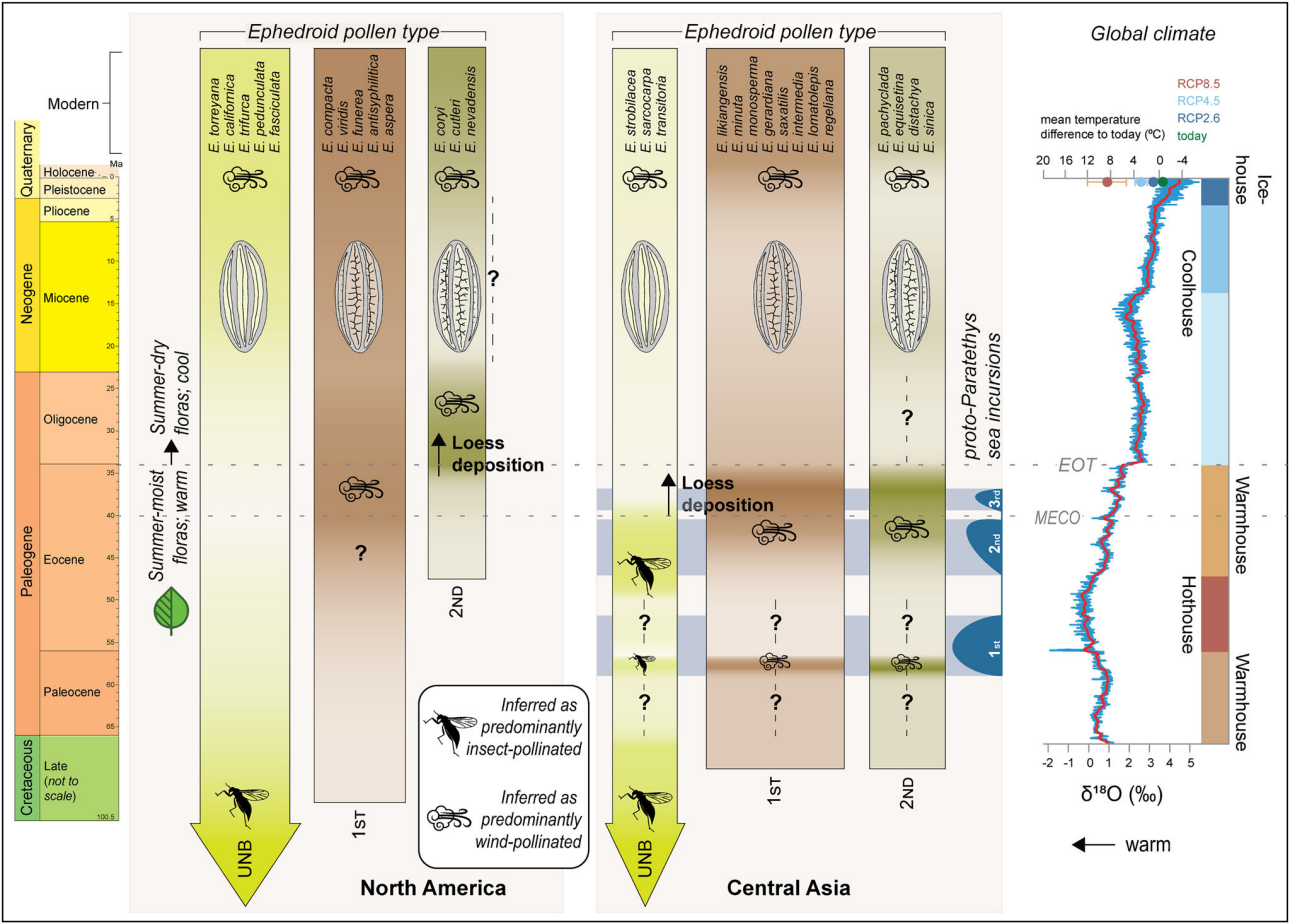
Evolution of ephedroid pollen types (depicted as shaded bars) in North America and Central Asia in relation to global temperatures (represented by the benthic foraminifera oxygen record of Westerhold *et al*. (2020) and Eocene–Oligocene aridity changes (represented by Proto‐Paratethys sea incursions; Kaya *et al*., 2019) in Central Asia and floristic turnover in Colorado (Leopold & Zaborac‐Reed, 2019). Future projections for global temperature in the year 2300 (Palmer, Harris & Gregory, 2018) are indicated by three representative concentration pathways (RCP) scenarios (light blue, dark blue, and orange circles) in comparison to today (green circle). UNB = unbranched (ancestral, type I) pollen grains; 1ST = pollen grains with first‐order branching of the pseudosulci (derived, type II); 2ND = pollen grains with first‐ and second‐order branching of the pseudosulci (derived, type III). Darker shading indicates increased abundance of that pollen type. Pollen abundances were compiled using references from Tables S2 and S3 as well as Jarzen *et al*. (2010), Loera *et al*., (2012), Garcia *et al*. (2016) and Han *et al*. (2016). Question marks indicate time periods of uncertainty. Modern biogeography of *Ephedra* species is from Bolinder *et al*. (2016b). EOT = Eocene–Oligocene Transition; MECO = Middle Eocene Climatic Optimum. Chronostratigraphy is from Cohen *et al*. (2013); updated to v2022/10).

The global, North American, and Central Asian palynological data sets synthesised here (Figs 1 and 6; Tables S2 and S3) document ephedroid diversity from the Cretaceous to the present, thus covering major climate changes over the K–Pg boundary (66 Ma), the Middle Eocene Climatic Optimum (MECO; 40 Ma) and the Eocene–Oligocene Transition (EOT; 34 Ma). We find clear evidence of substantial peaks and crashes in ephedroid diversity/abundance over time (Fig. 1) and the Cenozoic fluctuations involve differences in relative abundance of the various types of ephedroid pollen, differences that can be correlated with climate and pollination strategy (Fig. 6).

The presence of ephedroids in the Northern Hemisphere during the Early Cretaceous is clearly documented based on macrofossil as well as microfossil evidence (summarised, e.g. by Friis *et al*., 2011) but most records are from low palaeolatitudes where only pollen of the ancestral unbranched type is documented. At higher palaeolatitudes, presence of ephedroid pollen in rocks/sediments increases during the Late Cretaceous but is still mostly of the ancestral unbranched type. The eastern USA holds the earliest well‐documented record of the derived pollen of *Ephedripites* (Rydin *et al*., 2021). It is of the first‐order branching type (Fig. 3: type II), and was discovered by Steeves & Barghoorn (1959) from the upper part of the Raritan Formation [Zone V of Doyle & Robbins (1977) and personal communication with J.A. Doyle in 2021], which is of latest Turonian or Coniacian age (Massoni, Couvreur & Sauquet, 2015). Reports of the derived pollen type in older strata, for example of *Ephedripites fusiformis* from Cenomanian deep‐sea outcrops in the North Atlantic (Habib, 1970), exist but are extremely rare and need further research. In Asia, all three major types of ephedroid pollen (Fig. 3) are present by the latter Late Cretaceous (Fig. 6) and records of ephedroid pollen are abundant, with the ancestral unbranched forms such as *Ephedripites*, *Gnetaceaepollenites*, and *Steevesipollenites* the most common (as exemplified from the Xining Basin; Han *et al*., 2016).

In general, the ephedroid pollen record in North America and Central Asia follows the same trend; the ancestral type of pollen is dominant in the Cretaceous and derived pollen types become increasingly common in the Cenozoic (Fig. 6; Tables S2 and S3). However, while the derived first‐ and second‐order branched types seem to be equally prevalent in Central Asia, there is a transition from dominance of the first‐order branched type in the Middle Eocene (?Lutetian/Bartonian) to co‐dominance of both derived types at the Eocene–Oligocene Transition in North America (Fig. 6).

### (1) Climatic influences on North American ephedroids in the early–mid Cenozoic

To understand regional patterns of climate change and biodiversity change it is important to compare well‐dated records with ephedroid morphospecies‐level pollen records from several localities (Fig. 4). Such localities with good age control are rare (Barbolini *et al*., 2020), but are available for Eocene Central Asian basins such as the Xining Basin (see Section V.3 and Table S3). The poorer resolution for records in North America for localities containing abundant ephedroid pollen (Table S2) makes it more difficult to identify precisely which climatic drivers in that region were responsible for the increase in the Eocene of the derived first‐order branched pollen type. If this increase took place in the Bartonian, it may have been driven by reconfiguration of the hydrological cycle and atmospheric circulation after the MECO (Mulch *et al*., 2015; Methner *et al*., 2016). Alternatively, changing topography due to the southward growth of the North American Cordillera from 43 to 37 Ma (Mix *et al*., 2011; Chamberlain *et al*., 2012) resulted in drying and dust deposition in the central Cordillera (Fan *et al*., 2020). This atmospheric reconfiguration would have favoured the first‐order branched ephedroid pollen type presumed to be dispersed by wind, and thus could explain their increased abundance during the middle Eocene. From the latest Eocene, the second‐order branched type becomes the dominant form found while the first‐order branched type remains common (Fig. 6). By contrast, the ancestral (unbranched) pollen type is rare in these deposits, with only two species recorded over the late Eocene to late Oligocene (Fig. 6; Table S2).

The change to second‐order branched type predominating over the ancestral (unbranched) pollen type is coeval with the eastward expansion of a loess plateau in the western US driven by global cooling culminating in the EOT (Fig. 4A; Fan *et al*., 2020). Derived pollen remained dominant through the Oligocene in the western US (Table S2), during which the Chuska erg (a dune field of aeolian dust and sand in northwestern New Mexico and Arizona) continued to supply substantial amounts of airborne dust to the western US interior, until approximately 26 Ma (Cather *et al*., 2008; Cather, Chapin & Kelley, 2012). A substantial increase in aridity over the Eocene–Oligocene Transition reflected by floristic turnover (Leopold & Zaborac‐Reed, 2019), and an increase in the ratio of Pacific Ocean‐derived winter precipitation relative to Gulf of Mexico‐derived summer precipitation (Fan *et al*., 2020) likely also favoured the derived pollen types in North America.

### (2) Climatic influences on Central Asian ephedroids in the early‐mid Cenozoic

The onset of dust deposition and increases in aridity and winter precipitation also drove long‐term dominance of derived (branched pseudosulci) pollen in Central Asia, although it apparently started somewhat earlier in Central Asia than in North America (Fig. 6). Another difference is that derived pollen appears to dominate Central Asian environments for shorter intervals (<~1–2 million years) starting from the Late Paleocene – but does not remain dominant for longer periods until the mid‐late Eocene (earliest Bartonian; Table S3). At this point there is a spike in derived, second‐order branched ephedroid pollen in the Xining Basin (Table S3) which is likely coeval with a major environmental shift: the appearance of windblown dust in the sedimentary record, a change to predominant steppe‐desert vegetation, and the onset of obliquity‐dominated climate cyclicity which together indicate the onset of a high atmospheric pressure system driving intensified storms in the region (Bosboom *et al*., 2014; Meijer *et al*., 2021). As a key steppe‐desert plant, ephedras then remained consistently dominant in Central Asia for much longer than previously believed (~6 million years) until the EOT (Table S3; Barbolini *et al*., 2020). Our review shows that these ephedras belong almost exclusively to the group(s) producing derived pollen types (Table S3), that is wind‐pollinated species.

### (3) Climate change drives shifts in pollination syndrome

Evidence from the fossil record and investigations of living *Ephedra* species strongly indicate that a mix of pollination strategies existed in *Ephedra* since the Early Cretaceous, and that insect pollination was more common in this group in the past than it is today. Ephedroid pollen grains with branched pseudosulci, which as far as is known exclusively represent wind‐pollinated plants, are unknown prior to the Turonian–Coniacian (Late Cretaceous). However the unbranched ancestral pollen type, which is frequently present throughout the Cretaceous, is today produced by plants with several distinct pollination syndromes, insect‐pollinated plants (Bolinder *et al*., 2015, 2016, 2016), partly insect‐pollinated (ambophilous) plants (Meeuse *et al*., 1990; Celedón‐Neghme *et al*., 2016), and possibly also by some wind‐pollinated species (e.g. Niklas & Buchmann, 1987). Several distinct pollination syndromes are thus highly likely to have co‐existed in *Ephedra* throughout the evolutionary history of the group, with insect pollination possibly being significant during the Cretaceous and earliest Cenozoic.

The absence of sufficiently well‐dated sections for the early Paleocene of Central Asia (Fig. 6) makes it hard to judge precisely the effect of the K–Pg event on pollination and the biodiversity in this region. However, work conducted in North America as well as Central Asia (e.g. Wodehouse, 1933; Wang, Sun & Zhao, 1990a) found no evidence of a substantial decline in ephedroid pollen in the Northern Hemisphere in the aftermath of the K–Pg mass extinction. In fact, our review indicates the contrary, an increased occurrence of ephedroid pollen in the Northern Hemisphere that began in the latest Cretaceous and continued uninterrupted through the Paleocene (Fig. 1). In comparison, the age and palynoflora of Eocene records is better understood and particularly well‐studied for Central Asia where previous work clearly identified a general trend of branched ephedroid pollen grains successively replacing unbranched grains in the middle to late Eocene (Zhu *et al*., 1985; Han *et al*., 2016; Yuan *et al*., 2020).

Based on current scientific knowledge, we propose that the onset of dust storms in North America and Central Asia in the late Eocene marked a turning point in climate that was responsible for a long‐term sustained evolutionary shift in *Ephedra* where wind‐pollinated species successively became more common and wind pollination eventually the dominant pollination syndrome, as it is in *Ephedra* today. These climatic changes presumably led to an increased fitness of wind‐pollinated species and decreased fitness of insect‐pollinated species, as manifested in the observed increase in the derived types of ephedroid pollen and a concurrent decrease in the ancestral type (Fig. 6). Because the morphology of ephedran ovules and pollen grains is intricately linked to their reproductive strategy (see Fig. 2 and Section II), we argue that similar climatic changes in North America and Central Asia drove similar evolutionary changes in *Ephedra* during the Eocene (although slightly separated in time). This had permanent ramifications on ephedroid lineages, with pollen types with branched pseudosulci (produced only by wind‐pollinated plants) remaining dominant on both continents since the Oligocene and still today (Fig. 6).

In North America, a general increase in second‐order branched pollen coincides in time with the emergence of a North American loess plateau in the latest Eocene (Figs 4A and 6; Fan *et al*., 2020), and loess deposition expanded from the central Rocky Mountains eastward to the Great Plains during the EOT when the second‐order branched pollen type continued to increase (Fig. 6; Table S2). Similarly, the spike in derived, second‐order branched ephedroid pollen in Central Asia in the mid‐Eocene is coeval with a documented major environmental shift and the likely appearance of loess (dust) as evident from studies of the Xining Basin (Bosboom *et al*., 2014; Meijer *et al*., 2021). We consider loess as a reliable palaeoproxy for dust storms on both continents, because loess is typically preserved in foreland basins (Meijer & van der Meulen, 2023). Prior to the observed onset of loess in both North America and Central Asia, extensive and continuous foreland basin deposits are preserved that do not include loess. Therefore, if there was loess before, it would have accumulated in these basins.

The onset of loess is tightly linked to atmospheric reconfigurations in the late Paleogene (Bosboom *et al*., 2014; Meijer *et al*., 2021), which may have enhanced the efficiency of wind pollination and presented an adaptive advantage for wind‐pollinated ephedroids over insect‐pollinated species. Intensified winter/spring monsoonal dust storms would have facilitated long‐distance transport of pollen grains. Additionally, pseudosulcus branching increases the surface area of a pollen grain and reduces terminal settling velocity, improving pollen dispersal (Bolinder *et al*., 2015). This would have favoured plants producing derived types of pollen.

Furthermore, pseudosulci branching facilitates dehydration and subsequent rehydration of pollen grains (Bolinder *et al*., 2016b; Hofmann *et al*., 2022), which would be an advantageous feature for derived pollen types under the increasing aridity and more seasonal precipitation that took place in Central Asia after 40 Ma (Abels *et al*., 2011; Bosboom *et al*., 2014). However, this would trade off against a delay in germination of derived‐type grains, because pollen must rehydrate on the female structure first (Bolinder *et al*., 2016b). Regional cooling, aridification, and a shift towards more spring‐time precipitation at *ca*. 37 Ma (Dupont‐Nivet, Hoorn & Konert, 2008; Abels *et al*., 2011; Hoorn *et al*., 2012; Page *et al*., 2019) are linked with further increases in first‐ and second‐order branched taxa across multiple Central Asian basins (Xining, Nangqian, Qaidam) (Fig. 6; Table S3). If the Eocene (wind‐pollinated) derived types germinated in spring and the (insect‐pollinated) ancestral type germinated in summer as today (Bolinder *et al*., 2016a), a shift of the rainy season to cooler months would have advantaged wind‐pollinated plants by providing extended moisture during their germination period (Fig. 6).

Although wind pollination conceivably presented a distinct advantage over insect pollination when frequent dust storms and loess deposition began in the mid‐late Eocene, insect‐pollinated species probably co‐existed with wind‐pollinated species in many areas of the Northern Hemisphere at least until the Oligocene, as is the case still today in eastern Europe–western Asia. Co‐existence of wind pollination and insect pollination in ephedroids is particularly strongly indicated when both ancestral and derived pollen are detected, and by findings such as our discovery of fossil pollen of the ancestral type on a fossilised insect leg in Eocene sediments (Fig. 5A, B). The ancestral type of pollen grains produced by *E. foeminea* are today trapped by the setae of insect pollinators in exactly the same way (Fig. 5C; Bolinder *et al*., 2016a), suggesting the existence of a similar plant–pollinator mutualism during the Eocene.

An increased frequency of dust storms may also have disrupted reproduction in insect‐pollinated ephedras. Wind‐pollinated *Ephedra* today control the air flow around their ovules to the extent of being able to filter out allospecific pollen grains (Fig. 1; Niklas *et al*., 1986; Niklas & Buchmann, 1987), and presumably also dust particles (2–50 μm). Modern insect‐pollinated ephedras (*E. foeminea*; Bolinder *et al*., 2016a; Fig. 2C, F) do not control air flow around their ovules (Bolinder *et al*., 2015), so if this was also the case in the Eocene, dust could potentially have filled the pollination drop and blocked the exserted micropylar tube, preventing pollination. Notably, the insect‐pollinated *E. foeminea* today inhabits arid areas but without significant amounts of loess or windblown dust (GBIF, 2022). *Ephedra* and other Northern Hemisphere gymnosperms with upright ovules such as junipers and yews may have been particularly susceptible to wind‐blown dust, compared to, for example, pinaceous conifers, which have inverted ovules. The pollinators of *Ephedra* may also have been negatively affected by increasing aridity, but unfortunately Central Asian environments were not conducive to the preservation of insect fossils that could allow assessment of this. In order to survive the influx of dust and possible loss of pollinators, plants may instead have reproduced vegetatively (e.g. Land, 1913), explaining why the ancestral type of pollen did not immediately disappear as dust storms began (Fig. 6). Ultimately though, reliance on self‐propagation would have decreased plant fitness, which could explain the long‐term decline of the ancestral pollen type in the late Eocene, and the eventual dominance of wind pollination we see in *Ephedra* today.

### (4) Future perspectives

The Gnetales, to which *Ephedra* belong, have long been suggested to be an ancient plant group (e.g. Arber & Parkin, 1908), but fossil documentation of this group was sparse and poorly understood until relatively recently (Crane, 1996). A wealth of Early Cretaceous macrofossil evidence has been discovered in recent decades, and studies indicate that several innovations can likely explain how *Ephedra* has escaped extinction despite repeated diversity crises, among them a probable evolution of frost tolerance during the Cenozoic (K. Bolinder, A. Humphreys & C. Rydin, unpublished data), as well as the novelties and adaptations related to pollen structure and pollination discussed herein. It is now clear that ephedroids did not again become common in the equatorial region after the mid‐Cretaceous (Fig. 1; Crane & Lidgard, 1989) but several key factors and events in their long evolutionary history remain unclear and need further research.

One open question is why ephedroids are today confined to harsh environments such as steppes and deserts. They were clearly abundant in the equatorial region in the Early Cretaceous (Fig. 1) but never again comprised a substantial element of low (palaeo‐)latitude vegetation after a crash in the mid‐Cretaceous. Speculatively, they may have been unable to compete successfully with angiosperms, which were rapidly diversifying at that time (e.g. shown by palynological data in Crane & Lidgard, 1989). This should be studied further in the future, as should reasons for the apparent huge rise and fall of ephedroids in the Southern Hemisphere during the Late Cretaceous (Fig. 1).

The phylogeny of *Ephedra* remains unresolved. Despite a remarkable morphological stasis of ephedras since the Early Cretaceous (e.g. Rydin *et al*., 2004, 2006a; Rydin, Wu & Friis, 2006b), morphological, anatomical and histological work on female reproductive structures has indicated that the Cretaceous species are distantly related, extinct sister lineages to a much younger living clade (Rydin *et al*., 2010). Later work on pollen morphology tentatively suggested otherwise, that is that the living clade may be from the mid‐Cretaceous (Bolinder *et al*., 2016b), but further clarification is needed (see Rydin *et al*., 2021). The phylogeny and age of extant *Ephedra* has not been satisfactorily resolved despite extensive work based on genomic data (Rydin *et al*., 2021; R. Blokzijl, N. Wikström, L. Thilén & C. Rydin, in preparation); in particular, the deepest divergences in *Ephedra* are unclear and additional analyses, preferably based on genomic data, are needed. Nevertheless, there is some agreement between earlier studies based on molecular data (e.g. Huang & Price, 2003; Ickert‐Bond, Rydin & Renner, 2009; Loera, Sosa & Ickert‐Bond, 2012) and our results based on microfossil data. For example, we show here that the appearance of derived (branched) ephedroid pollen was likely the result of similar evolutionary pressures in Asia and North America, rather than a shared evolutionary history, and that the second‐order derived pollen type appears earlier in Asia than in North America, which is consistent with results of phylogenetic analyses (e.g. Rydin *et al*., 2004, 2021; Ickert‐Bond *et al*., 2009; Rydin & Korall, 2009).

Because the post‐Early Cretaceous history of *Ephedra* is so far only directly documented from microfossils, additional studies of ephedroid pollen and investigations of what it can and cannot tell us about the ecology of the plant that produced it are of highest relevance. A key question is whether fossil pollen preserves decisive information on pollination biology. Current knowledge strongly suggests that the derived pollen type is produced exclusively by wind‐pollinated plants, whereas the ancestral pollen type may be linked to several pollination syndromes. Ecological field studies of additional extant species and further studies of pollen wall properties and terminal settling velocity of extant and fossil pollen grains may provide clarity on this.

## VI. CONCLUSIONS

(1) Ephedroids are ancient survivors that have successfully endured ever‐increasing competition from diversifying angiosperms and periods of severe stress caused by abiotic factors such as the end‐Cretaceous extraterrestrial impact and the dramatic change from a greenhouse world to an icehouse world in the Oligocene. They have constituted an important element in the vegetation at least until the earliest Oligocene, for example in pre‐grassland open habitats of mid‐palaeolatitudes of the Northern Hemisphere, and continue to do so in highly arid areas of the world.
(2) A mix of pollination strategies is suggested to have occurred in *Ephedra* at least since the mid‐Late Cretaceous, probably since the Early Cretaceous. Insect pollination was more common in *Ephedra* in the Cretaceous than today. The ancestral type of *Ephedra* pollen with unbranched pseudosulci is today produced by insect‐pollinated species, ambophilous species and probably also by some wind‐pollinated species. The derived type of *Ephedra* pollen with branched pseudosulci, first documented from mid Late Cretaceous North American strata, became increasingly common in the early–mid Cenozoic. This pollen type is today produced exclusively by wind‐pollinated plants. The trend towards wind pollination being adopted as the dominant pollination syndrome in *Ephedra*, as it is today, can likely be explained by an increased fitness of wind‐pollinated species as well as decreased fitness of insect‐pollinated species following early Cenozoic climatic changes.
(3) We propose that rapid changes in temperature, aridity and seasonality strongly influenced the type of ephedroid pollen detected in sedimentary archives, and likely the reproductive strategy of these ancient gymnosperms. Early Cenozoic climate changes in Asia and North America that resulted in more frequent dust storms appear to have selected for ephedroid species with the derived pollen type (i.e. wind‐pollinated species) at the expense of species that produced the ancestral pollen type (i.e. species that relied entirely or partly on insects for pollen transfer and possibly also anemophilous species). Additional studies of fossil ephedroid pollen and the information on pollination syndrome it may have preserved would be highly valuable.
(4) The long‐term, well‐dated ephedroid microfossil record in the Xining Basin (China) enables a correlation to be detected between ephedroid pollen type and climatic changes. Similar Cenozoic climatic changes in North America and Central Asia triggered similar morphological and evolutionary responses in *Ephedra*. It is clear that grouping fossil ephedras by pollen pseudosulcus branching type has validity for understanding past climate, vegetation and ecology. Pollen pseudosulcus branching type, which is preserved in the fossil record, is an indicator of past plant ecological preferences and pollination strategies. Our results from North America mimic the patterns detected for Central Asia; climate‐related fluctuations in the relative frequencies of different pollination strategies follow the same trends in these vastly separated locations.
(5) *Ephedra* may provide a precedent showing how large‐scale temperature changes and reconfiguration of atmospheric systems can drive long‐term changes in pollination type. Today, desertification is spreading across Asia (and other continents) at rapid rates, and major changes are already evident in the modern East and South Asian monsoons (IPCC, 2023). Similar climatic changes had permanent ramifications for pollination biology and reproduction of *Ephedra* in the past: a valuable lesson for predictions on how plant–pollinator networks may be affected by current and future global change.

## Supporting information


**Table S1.** Global distribution of ephedroids through time.

**Table S2.** North American ephedroid palaeopalynomorphs.

**Table S3.** Central Asian ephedroid pollen data.
